# Effects of feeding a *Bacillus*-based probiotic on feed intake, performance, carcass traits, and health of crossbred beef cattle fed sodium monensin and high-moisture corn-based diets during finishing

**DOI:** 10.1093/tas/txag124

**Published:** 2026-08-07

**Authors:** Juan Bollatti, Federico Bernardi, Ramiro Andreola, Fernando M Clemente, Oscar C M Queiroz, Reinaldo F Cooke, Bruno I Cappellozza

**Affiliations:** Biofarma Experimental Center for Animal Nutrition, Sinsacate X5223, Argentina; Biofarma Experimental Center for Animal Nutrition, Sinsacate X5223, Argentina; Biofarma Experimental Center for Animal Nutrition, Sinsacate X5223, Argentina; Novonesis, Buenos Aires C1107, Argentina; Novonesis, Lyngby 2800, Denmark; Department of Animal Science, Texas A&M University, College Station, TX 77843, United States; Novonesis, Lyngby 2800, Denmark

**Keywords:** direct-fed microbial, fever, grain processing, hot carcass weight, sodium monensin

## Abstract

This experiment evaluated feed intake, performance, fecal nutrient concentrations, health, and carcass traits of crossbred beef cattle offered a high-moisture corn-based diet containing sodium monensin. On day 0 of the study, 402 crossbred steers [initial body weight (*BW*) 329 ± 69.5 kg] were blocked by BW and source into 1 of 28 feedlot pens (14 to 15 steers/pen). Within blocks, pens were assigned to high-moisture corn-based diets containing (*BAC*) or not (*CON*; *n* = 14) *Bacillus licheniformis* 809 and *B. subtilis* 810 (2 g/steer per day; *n* = 14). The experimental period lasted 118 days and BAC was added to a mineral-vitamin premix, which also contained sodium monensin. Throughout the experimental period, dry matter intake was determined daily, while individual BW was obtained on days 0, 35, and 118 of the study. Based on these measurements, average daily gain (*ADG*) and gain to feed (*GF*) were also calculated. Fecal samples were collected on days 20 and 70 of the study for determination of fecal starch and nitrogen (*N*). On day 122, animals were transported to a commercial slaughterhouse and carcass traits were determined. All data were analyzed with SAS (version 9.4) using pen as the experimental unit. Body weight on days 35 and 118 was greater (*P* ≤ 0.03) for BAC vs. CON, while no changes were observed on feed intake (*P* ≥ 0.40). Average daily gain was also greater for BAC from days 0 to 35 (*P* = 0.01) and 0 to 118 (*P* = 0.03). Hence, GF was improved by 8.4 and 2.9% when assessed from days 0 to 35 (*P* = 0.03) and 0 to 118 (*P* = 0.04), respectively. Hot carcass weight was also greater for BAC (+ 3.5 kg; *P* = 0.05), while *Biceps femoris* fat thickness tended to be greater (*P* = 0.07) for BAC. Carcass-adjusted final BW, ADG, and GF were also greater (*P* ≤ 0.05) for BAC. No differences were observed on fecal starch and N (*P* ≥ 0.42), but fecal score tended to be greater for BAC from days 35 to 118 and from days 0 to 118 (*P* ≤ 0.09). No treatment effects were detected for the incidence of bovine respiratory cases (*P* ≥ 0.12), while overall morbidity tended to be lower (*P* = 0.06) and fever occurrence in steers showing bovine respiratory disease was lower in steers fed BAC (*P* = 0.04). In summary, *Bacillus* spp. supplementation optimized performance, health, and carcass traits of feedlot cattle fed a high-moisture corn-based diet containing sodium monensin.

## Teaser

This study reported the benefits of *Bacillus*-based probiotics on performance, carcass characteristics, and health of fattening beef cattle offered a high-starch diet containing sodium monensin.

## Introduction

Feedlot diets often contain considerable amount of cereals (Vasconcelos and Galyean 2007; Samuelson et al. 2016), aiming to optimize overall live performance, carcass characteristics, and profitability of the operations. In addition to feeding a high proportion of cereals in the finishing diet, nutritionists are looking for strategies that increase the availability and, hence, the utilization of starch in the rumen (Vasconcelos and Galyean 2007; Samuelson et al. 2016; Monsalve and Millen 2025), including high-moisture corn. As an example, previous authors reported an improvement in starch utilization, lower fecal starch, and greater feedlot average daily gain (*ADG*) and feed efficiency (*FE*) when corn grain was processed (Vander Pol et al. 2008; Owens et al. 2016). Nonetheless, these diets, although needed in the production systems, may also challenge the function and health of the rumen by causing acidosis, overall health of the animals, as well as its ruminal microbiota (Gozho et al. 2005; Khafipour et al. 2009, 2016). Hence, feed additives play an important role in maintaining rumen and overall health, while optimizing performance of feedlot cattle fed high-starch diets.

*Bacillus*-based probiotics have been extensively reviewed in a recent publication (Cappellozza et al. 2025), but improvements on feed efficiency have been reported in feedlot cattle consuming finishing diets containing 33% to 50% starch (Dias et al. 2022; Cordeiro et al. 2024). Among the potential modes of action underlying these mechanisms, enzyme production (Luise et al. 2022; Schallmey et al. 2004), nutrient digestion (Limede et al. 2025; Souza et al. 2026), modulation of rumen fermentation (Souza et al. 2026), and overall health benefits (Mackey et al. 2024, 2025) might explain these results. More specifically, *Bacillus* spp. stimulate biofilm formation (Segura et al. 2020) and mucin production in intestinal epithelial cells (Santano et al. 2020), alleviate the occurrence of the hyperpermeable intestine syndrome when exposed to reactive oxygen species (Boll et al. 2024), maintain the balance of the microorganisms in the gut (Capern et al. 2026), and modulate the immune response following different challenges (Mackey et al. 2025). However, to our knowledge, no other study evaluated the effects of *Bacillus* spp. supplementation on feedlot diets based on high-moisture corn and supplemented with sodium monensin, an ionophore known to modulate rumen fermentation and feed efficiency in feedlot cattle (Duffield et al. 2012; Marques and Cooke 2021). Therefore, our objective was to evaluate the effects of *Bacillus* spp. supplementation on feed intake, growth rates, and carcass traits of crossbred cattle offered a high-moisture corn based finishing diet containing sodium monensin.

## Materials and methods

This experiment was conducted at the Experimental Center for Animal Nutrition—Biofarma (Jesús Maria, Cordoba, Argentina) from October 2025 to February 2026. All animals were cared for in accordance with the practices outlined in the Guide for the Care and Use of Agricultural Animals in Agricultural Research and Training (FASS 2020) and managed following accepted practices and experimental protocols, approved by the Animal Ethical Use Committee of National University of the Litoral, Faculty of Veterinary Sciences, Santa Fe, Argentina (approval no. FCV-1278961-26).

### Animals, housing, and treatments

All steers used in the current experiment were sourced from commercial operations (*n* = 8) and transported to the research site at least 3 days prior to the beginning of the experimental period. On day 0 of the study, 402 crossbred (Angus, Hereford, Brangus, and Braford) beef steers [initial body weight (*BW*) 329 ± 69.5 kg] were blocked by source and initial BW and assigned to 1 of 28 feedlot pens (8 × 20 m). Within block, pens were randomly assigned to: (1) high-moisture corn-based diet (*CON*; *n* = 14) or (2) CON with the addition of a *Bacillus*-based probiotic containing *Bacillus licheniformis* 809 and *B. subtilis* 810 (2 g/head per day; Bovacillus, Novonesis, Buenos Aires, Argentina; 3.2 × 10^9^ CFU/g; *BAC*; *n* = 14). No previous information regarding the management and health records were obtained from the animals prior to the enrollment of the study. The experimental period lasted 118 days and was split into three total mixed ration (*TMR*) adaptation diets (days 0 to 7, 8 to 14, and 15 to 21, respectively), followed by a finishing diet offered from days 22 to 118. Diets were offered twice a day at equal amounts, allowing 5% of orts in the following day. The BAC was offered throughout the experimental period, while both CON and BAC received sodium monensin at a rate of 36 mg/kg. A complete composition and description of the experimental diets is reported in Table 1, while Table 2 presents the particle size and distribution of the high-moisture corn, cracked corn, as well as the corn and sorghum silages. To prevent cross-contamination between treatments, diets for CON pens were always mixed and offered prior to BAC diets. Following BAC diet preparation and delivery, the mixer wagon was thoroughly cleaned by mixing and circulating a flush batch of silage, which was subsequently discarded and excluded from this study. On day 0 of the study, all animals received vaccines against clostridial diseases (5 mL/head; Providean Clostridial 10P; Tecnovax, Buenos Aires, Argentina), rabies (2 mL/head; CDVac Rabia; CDV, Buenos Aires, Argentina) and were administered ricobendazole (2 mL/40 kg BW; Bayverm PI; Elanco Animal Health, Indianapolis, IN) as anthelmintic, and ivermectin (1 mL/50 kg BW; Vermectín L.A. Premium; Over, Santa Fe, Argentina) for the control of ectoparasites.

**Table 1:** Composition and nutritional profile of the total mixed rations (*TMR*) offered throughout the current study^a^.

Item	ADAP-1	ADAP-2	ADAP-3	FIN
**Composition, DM basis**				
**High moisture corn, %**	12.0	24.0	35.0	48.0
** Cracked corn, %**	18.0	17.8	21.0	20.0
** Sorghum and corn silage^b^, %**	20.0	16.0	8.0	7.0
** Alfalfa hay, %**	25.0	18.0	12.0	4.0
**Wet distillers grains, %**	20.0	20.0	20.0	18.0
** Vitamin-mineral mix^c^, %**	5.0	4.2	4.0	3.0
**Dry matter, % as is**	52.1	55.7	54.8	55.1
**Nutritional profile,^d^ DM basis**				
** Net energy for maintenance, Mcal/kg**	1.70	1.84	1.98	2.11
** Net energy for gain, Mcal/kg**	1.09	1.21	1.34	1.45
** Total digestible nutrients, %**	70.4	74.8	79.4	83.4
** Neutral detergent fiber, %**	32.5	30.2	26.9	20.9
** Acid detergent fiber, %**	20.3	16.4	14.5	10.5
** Ash, %**	7.5	7.2	5.9	5.3
** Crude protein, %**	16.7	15.9	15.1	15.0
** Ether extract, %**	3.6	4.2	4.2	4.0
** Starch, %**	27.3	34.8	43.2	51.5
** Non-fiber carbohydrates, %**	39.7	42.4	47.8	54.8
**Particle size distribution,^e^ % total**				
** > 8 mm**	8.0	6.2	3.9	2.2
** 8 to 4 mm**	30.2	27.3	21.4	20.0
** < 4 mm**	61.8	66.5	74.7	77.8

a *ADAP-1*,  adaptation diet offered from days 0 to 7; *ADAP-2,  *adaptation diet offered from days 8 to 14; *ADAP-3,  *adaptation diet offered from days 15 to 21; *FIN*,  finishing diet offered from days 22 to 118.

b Sorghum and corn silage were mixed and added in the diets at a 55:45 ratio (DM basis).

c Containing 20% Urea, 18% Ca, 22% NaCl, 370 mg/kg Cu, 4 mg/kg Co, 8 mg/kg Se, 1625 mg/kg Zn, 1322 mg/kg Mn, 18 mg/kg I, 105,000 IU/kg of vitamin A, 21,000 IU/kg of vitamin D3, and 140 IU/kg of vitamin E, 1200 mg/kg of monensin (36 g/ton of total mixed ration, dry matter basis), and 160 mg of diflubenzuron (Biofarma, Argentina).

d Based on wet chemistry procedures by a commercial laboratory (Feedlab Biofarma). Net energy for maintenance (NEm = 1.37 × ME—0.138 × ME² + 0.0105 × ME³—1.12), net energy for gain (NEg = 1.42—0.174 × ME² + 0.0122 × ME³—1.65), and non-fiber carbohydrates were calculated using the equations described by NASEM et al. (2016).

e Performed as described by Kononoff and Heinrichs (2003).

**Table 2:** Particle size of the different ingredients used in the current experiment.

*Pores in the sieve, % of total*		*High-moisture corn^a^*	*Cracked corn^a^*
** > 6.0 mm**		9.1	0.9
** ≤ 6.00 and > 3.5 mm**		13.4	2.8
** ≤ 3.50 and > 2.0 mm**		24.8	57.1
** ≤ 2.00 and > 1.25 mm**		35.8	23.5
** ≤ 1.25 mm**		16.8	15.7
**Mean particle size of corn, mm^b^**		2.74	2.26
**Geometric mean diameter (dgw), mm^c^**		2.05	1.92
***Pores in the sieve,^d^ % of total***		***Sorghum and corn silage^e^***	***Alfalfa hay^e^***
** > 8 mm**		11.5	30.5
** 8 to 4 mm**		58.4	23.6
** < 4 mm**		30.1	45.9

a Dry matter content was 70.3 and 90.4%, respectively, for high-moisture corn and cracked corn.

b Corn retained on the 6 mm screen was determined in 20 randomly particles using a digital caliper. The residue retained in the bottom was assumed to have a mean particle size of 0.625 mm. Based on Yu et al. (1998).

c Calculated as geometric mean diameter (dgw) assuming a log-normal particle size distribution according to ASAE Standard S319.3 [dgw = exp(Σ pi × ln $\bar{\text{d}}$i)], where pi represents the mass fraction and $\bar{\text{d}}$i the mean diameter of sieve i.

d Performed as described by Kononoff and Heinrichs (2003).

e Dry matter content was 30.2 and 88.1%, respectively, for the mixture of sorghum and corn silage and alfalfa hay.

### Sampling, laboratorial analyses, and health observations

Samples of the TMRs were taken at the beginning of each diet offer and stored at −20°C until further laboratorial analyses. All samples were dried in a forced-air circulation oven (IS2300; FAETA; Buenos Aires, Argentina) at 55°C for 72 h and then ground in a Wiley mill (Wiley TE-680, Philadelphia, United States). After milling, the absolute DM concentration was obtained by drying in a laboratory oven at 105°C (method 934.01; AOAC 1990) and analyzed for ash (4 h at 550°C; method 642.05; AOAC 1995), CP by the Kjeldahl method (method 988.05; AOAC 1995), NDF according to Van Soest et al. (1991) using sodium sulfite and alpha-amylase, and ether extract using a Soxhlet apparatus after an acid hydrolysis preparation (method 942.05 AOAC 1995). Starch was determined according to the individual values of the feedstuffs, while net energy for maintenance (*NE_m_*), net energy for gain (*NE_g_*), and total digestible nutrients (*TDN*) were determined according to equations described in NASEM (2016). Lastly, non-fiber carbohydrates (*NFC*) were determined as follows: NFC = 100 – (CP + NDF + ether extract + ash).

Individual BW was collected on days 0, 35, and 118 of the study. Body weight measurements collected on days 0 and 118 were obtained after 24 h of feed withdrawal with 4% pencil-shrink calculation (BW × 0.96; NASEM 2016), while the BW on day 35 was collected after 16 h of feed withdrawal and 6% pencil-shrink was applied to the measured BW (BW × 0.94; Coffey et al. 2001). Then, average daily gain (*ADG*) was calculated from days 0 to 35, 36 to 118, and from days 0 to 118. Throughout the experimental period, feedbunks were managed daily, with orts weighed and sampled for DM only when accumulation exceeded 10% of the daily offer or during the scheduled weekly bunk cleanouts. Dry matter of the offered and orts were determined by drying the samples at 100°C for 24 h. Gain to feed ratio (*GF*) was then determined by dividing the ADG by the pen DMI and reported as kg gain/kg DM consumed. Moreover, the observed net energy (*NE*) for each diet was calculated from the performance data using the equations reported by Zinn and Shen (1998) based on pen average values. Energy gain (*EG*) was calculated as EG = (0.0557 × BW^0.75^) × ADG^1.097^ (NRC 1984), in which EG is daily energy deposited (Mcal/d) and BW is the mean shrunk BW. No further correction for frame size was performed herein, as the medium-frame steer equation was used, assuming 28% empty body fat at 478 kg of BW. The equation used to calculate maintenance energy expended (MEx; Mcal/d) was MEx = 0.077 × BW^0.75^ (NRC 1996). From the calculated amounts of energy required for maintenance (NE_m_) and gain (NE_g_), the NE_m_ of each diet was obtained by the quadratic equation NE_m_ = [−*b* ± (*b*2 − 4*ac*)1/2]/2*a*, in which **a** = − 0.877 × DMI, **b** = 0.877 × MEx + 0.41 × DMI + EG, and **c** = − 0.42 × MEx and NEg of each diet was obtained by the equation NE_g_ = 0.877 × NE_m_ − 0.41 (Zinn and Shen 1998).

Every week throughout the experimental period, fecal score was determined in each pen assigned to the study, where 1 = liquid feces with blood, 2 = liquid feces, 3 = semi-hard feces, 4 = hard feces, and 5 = extremely hard feces (Ireland-Perry and Stallings 1993). Fecal samples were collected twice, on days 20 and 70 of the study, over two consecutive days and twice a day (approximately at 0900 and 1400 h). hTis sampling scheme was adopted to represent the fecal starch and N levels of the adaptation and finishing diets offered during the study. In each sampling, fecal samples of five random animals (50 g/animal) were collected from the floor of each pen immediately after defecation. Fecal samples were pooled and then stored at −20°C for subsequent analysis of fecal starch and N. Fecal starch was determined using the K-TSTA kit (Megazyme, Lansing, MI, USA; method 996.11; AOAC 1986), while fecal N was determined according to the Kjeldahl method (method 988.05; AOAC 1995).

Steers were observed daily by the same trained observer for the occurrence of adverse health events. Any animal that required a pharmacological intervention also had its rectal temperature assessed. For bovine respiratory disease (*BRD*) occurrence, clinical signs, including depression (lethargy, drooping ears), abnormal posture (gauntness, orthopnea), and altered respiratory behavior (nasal discharge, dyspnea) were observed. Steers diagnosed with BRD signs received a dose of meloxicam (s.c.; 0.5 mg/kg BW; Metacam, Boehringer Ingelheim, Ingelheim, Germany) and were assessed for rectal temperature. Steers classified as febrile (rectal temperature > 39.5°C) also received tulathromycin (Draxxin; Zoetis; Buenos Aires, Argentina) at 2.5 mg/kg of BW subcutaneously as their first therapeutic antimicrobial treatment. Treatment failure was defined as animals requiring a second treatment within 14 d of the initial therapy. In such cases, florfenicol (s.c.; 40 mg/kg BW; Nuflor, Merck Animal Health, Madison, NJ) was administered as the second-line antimicrobial treatment. Steers diagnosed with BRD signs after the second antimicrobial treatment received enrofloxacin only (s.c.; 7.5 mg/kg BW; Baytril, Bayer Animal Health, Shawnee Mission, KS). None of the steers required a fourth treatment and/or died during the current experiment.

### Carcass measurements

On day 110 of the experimental period, all animals were subjected to ultrasound evaluations (ProVetScan SU-3A with an 18.0 cm/3.50 MHz linear probe; New Vetec S. L., León, Spain), performed by the same trained technician. Evaluations were conducted according to procedures described by the Ultrasound Guidelines Council (UGC 2012) and measurements of the *Longissimus muscle* ribeye area (cm^2^), backfat thickness (mm), and *Biceps femoris* fat thickness (mm) were collected.

All animals were slaughtered on the morning of day 122 following a waiting period of approximately 16 h, in a commercial packing plant. Hot carcasses were separated into two symmetrical sections, weighed to obtain hot carcass weight (*HCW*), and individually identified. Dressing percent was calculated by dividing the HCW and final BW of each animal. Based on these evaluations, final BW was determined based on HCW, while carcass ADG and FCR were calculated using a dressing percentage of 58%, according to industry guidelines in Argentina. Carcass-adjusted ADG was determined by the difference of carcass-adjusted final BW and initial BW, divided by the number of days in the study (118 days), while carcass-adjusted FCR was calculated as DMI divided by carcass-adjusted ADG.

### Statistical analysis

All continuous data were analyzed using the MIXED procedure of SAS (version 9.4; SAS Institute Inc., Cary, NC) with pen as the experimental unit. The statistical model included treatment as a fixed effect and block as a random effect. For growth performance, feed intake, and carcass characteristics, initial BW was included as a continuous covariate when appropriate. Significance was declared at *P* ≤ 0.05, whereas tendencies were denoted if 0.05 < *P* ≤ 0.10. Fecal nutrient content (N and starch) was also tested as a repeated measurement, using treatment, day, and the resulting interaction as fixed effects, day as a repeated factor, and the covariance matrices were tested (autoregressive-1, unstructured, compound symmetry, and Toeplitz). However, no trends or significances (*P* ≥ 0.10) were noted and, therefore, only the main effects of each day will be reported. The GLIMMIX procedure of SAS (SAS Inst. Inc.) with a binomial distribution and logit link function was used to analyze health responses (BRD incidence, fever). The model was fitted using an events/trials syntax, where the number of affected animals within a pen was defined as the event and the initial number of animals enrolled in that pen was the number of trials. The model specified a binomial distribution and a logit link function. The statistical model included the fixed effect of treatment and the random effect of block and pen. The Newton-Raphson optimization technique was applied to aid model convergence, and denominator degrees of freedom were approximated using the Kenward-Roger method. Least squares means and their respective standard errors were back-transformed to the original data scale (proportions) using the inverse link function (ILINK). Assumptions of normality and homoscedasticity of studentized conditional residuals were confirmed via the Shapiro-Wilk test (*P* > 0.10) and visual inspection of residual vs. predicted value plots. Observed pen-level incidence rates remained well within distribution boundaries, ensuring the suitability of the linear mixed model. Lastly, the temporal dynamics of disease occurrence (BRD, general morbidity, and BRD with confirmed fever) were evaluated using non-parametric Kaplan-Meier survival analysis (PROC LIFETEST of SAS). Differences between treatment curves across the 118-d feeding period were evaluated using Log-Rank and Wilcoxon tests. Because cumulative incidence remained below 50% across treatments, median time-to-event was unreached; thus, the 25th percentile of cumulative incidence and Restricted Mean Survival Time (*RMST*) were reported. Results are expressed as least squares means and standard errors of the mean. All results are presented as least square means and separated using the LSD.

## Results

### Feedlot performance

Table 3 reports the live performance of the animals during the study. No differences were observed on initial BW (*P* = 0.49), while treatment effects were detected for BW on days 35 and 118 of the study (*P* ≤ 0.03). Steers fed BAC were 4.0 and 6.0 kg heavier than CON cohorts, respectively (*P* ≤ 0.03). In agreement, ADG from days 0 to 35 and from days 0 to 118 were also greater (*P* ≤ 0.03) for BAC vs. CON, while no differences were noted on ADG from days 36 to 118 (*P* = 0.35). No treatment effects were observed for DMI (*P* ≥ 0.40) at any period of the experiment; hence, GF was 8.4 and 2.9% greater for BAC from days 0 to 35 and from days 0 to 118, respectively (*P* ≤ 0.04), but no treatment effects were observed on GF from days 36 to 118 (*P* = 0.61).

**Table 3:** Performance results of crossbred beef steers receiving a high-moisture corn-based diet containing or not (*CON*; *n* = 14) a *Bacillus*-based probiotic in the diet (*BAC*; *n* = 14)^a^^,^^b^.

Item	Treatments		SEM	*P-*value
	CON	BAC		
**Body weight, kg**				
** Initial**	329	329	18.2	0.49
** Day 35**	388	392	3.0	0.01
** Final (day 118)**	482	488	6.7	0.03
**Average daily gain, kg**				
** Days 0 to 35**	1.58	1.69	0.07	0.01
** Days 36 to 118**	1.17	1.19	0.05	0.35
** Days 0 to 118**	1.30	1.35	0.05	0.03
**Dry matter intake, kg/day**				
** Days 0 to 35**	9.57	9.51	0.22	0.70
** Days 36 to 118**	9.65	9.73	0.32	0.40
** Days 0 to 118**	9.62	9.66	0.28	0.70
**Gain to feed, kg/kg**				
** Days 0 to 35**	0.166	0.180	0.006	0.03
** Days 36 to 118**	0.122	0.123	0.002	0.61
** Days 0 to 118**	0.136	0.140	0.003	0.04

a Treatments were offered daily from days 0 to 118. The BAC contained 2 g of *Bacillus licheniformis* 809 and *B. subtilis* 810 (6.4 × 10^9^ CFU/head per day; Bovacillus, Novonesis, Buenos Aires, Argentina) and was added to the mineral-vitamin premix.

b Sodium monensin was added to the mineral-vitamin premix of both treatments throughout the experimental period to ensure a dosage of 36 mg/kg.

Table 4 reports the energy intake (NE_m_ and NE_g_), as well as observed, expected, and the observed to expected ratio energy values of the diets. In accordance with the DMI results, no treatment effects were observed on NE_m_ or NE_g_ intake in the different phases of the study (*P* ≥ 0.40). Nonetheless, treatment effects were reported on observed NE_m_ and NE_g_ from days 0 to 35 and 0 to 118 (*P* ≤ 0.04), as BAC had greater values for both variables. Hence, the observed to expected NE_m_ and NE_g_ ratios were greater for BAC vs. CON (*P* ≤ 0.04) on the same timepoints as mentioned above.

**Table 4:** Dietary energy intake and efficiency of energy utilization of crossbred beef steers receiving a high-moisture corn-based diet containing or not (*CON*; *n* = 14) a *bacillus*-based probiotic in the diet (*BAC*; *n* = 14)^a^^,^^b^.

Item	Treatments		SEM	*P-*value
	CON	BAC		
**NE_m_ intake, Mcal/d**				
** Days 0 to 35**	19.05	18.96	0.43	0.77
** Days 36 to 118**	20.37	20.53	0.67	0.40
** Days 0 to 118**	19.94	20.02	0.58	0.65
** NE_g_ intake, Mcal/d**				
** Days 0 to 35**	12.87	12.82	0.29	0.79
** Days 36 to 118**	14.00	14.11	0.46	0.40
** Days 0 to 118**	13.64	13.69	0.39	0.64
**Expected NE_m_, Mcal/kg**				
** Days 0 to 35**	1.99	1.99	0.01	0.23
** Days 36 to 118**	2.11	2.11	—	—
** Days 0 to 118**	2.07	2.07	0.002	0.21
**Expected NE_g_, Mcal/kg**				
** Days 0 to 35**	1.35	1.35	0.01	0.22
** Days 36 to 118**	1.45	1.45	—	—
** Days 0 to 118**	1.42	1.42	0.002	0.21
**Observed NE_m_,^c^ Mcal/kg**				
** Days 0 to 35**	1.86	1.96	0.05	0.03
** Days 36 to 118**	1.77	1.79	0.02	0.45
** Days 0 to 118**	1.79	1.83	0.02	0.04
**Observed NE_g_,^c^ Mcal/kg**				
** Days 0 to 35**	1.22	1.31	0.04	0.03
** Days 36 to 118**	1.14	1.16	0.02	0.45
** Days 0 to 118**	1.16	1.20	0.02	0.04
**Observed: Expected NE_m_^d^**				
** Days 0 to 35**	0.94	0.98	0.02	0.04
** Days 36 to 118**	0.84	0.85	0.01	0.45
** Days 0 to 118**	0.87	0.88	0.01	0.04
**Observed: Expected NE_g_^d^**				
** Days 0 to 35**	0.91	0.97	0.03	0.03
** Days 36 to 118**	0.79	0.80	0.01	0.45
** Days 0 to 118**	0.82	0.84	0.01	0.04

a Treatments were offered daily from days 0 to 118. The BAC contained 2 g of *Bacillus licheniformis* 809 and *B. subtilis* 810 (6.4 × 10^9^ CFU/head per day; Bovacillus, Novonesis, Buenos Aires, Argentina) and was added to the mineral-vitamin premix.

b Based on wet chemistry procedures by a commercial laboratory (Feedlab Biofarma). Net energy for maintenance (NEm = 1.37 × ME—0.138 × ME² + 0.0105 × ME³—1.12), net energy for gain (NEg = 1.42—0.174 × ME² + 0.0122 × ME³—1.65), and non-fiber carbohydrates were calculated using the equations described by NASEM et al. (2016).

c Net energy for maintenance and gain based on performance were calculated based on Zinn and Shen (1998).

d For the observed over expected NE, expected NE was calculated using tabular data (NASEM et al. 2016).

### Fecal sampling

Table 5 reports the fecal score and fecal content of N and starch. No treatment effects were observed on the fecal score between day 0 and 35 (*P* = 0.18), while it tended to be 2.6 and 2.2% greater for BAC vs. CON from days 35 to 118 and from days 0 to 118 (*P* ≤ 0.09), respectively. Regarding fecal starch and N contents, no differences were observed either on days 20 or 70 of the study (*P* ≥ 0.42).

**Table 5:** Fecal score, starch, and N levels of crossbred beef steers receiving a high-moisture corn-based diet containing or not (*CON*; *n* = 14) a *bacillus*-based probiotic in the diet (*BAC*; *n* = 14)^a^^,^^b^.

Item	Treatments		SEM	*P-*value
	CON	BAC		
**Fecal score^c^**				
** Days 0 to 35**	2.76	2.81	0.06	0.18
** Days 36 to 118**	2.69	2.76	0.03	0.09
** Days 0 to 118**	2.72	2.78	0.04	0.08
**Fecal starch,^d^ % dry matter**				
** Day 20**	11.5	10.4	0.85	0.42
** Day 70**	9.42	9.42	0.39	1.00
**Fecal N,^d^ % dry matter**				
** Day 20**	2.30	2.30	0.03	0.91
** Day 70**	2.27	2.29	0.04	0.55

a Treatments were offered daily from days 0 to 118. The BAC contained 2 g of *Bacillus licheniformis* 809 and *B. subtilis* 810 (6.4 × 10^9^ CFU/head per day; Bovacillus, Novonesis, Buenos Aires, Argentina) and was added to the mineral-vitamin premix.

b Sodium monensin was added to the mineral-vitamin premix of both treatments throughout the experimental period to ensure a dosage of 36 mg/kg.

c Fecal score was assessed in each pen, where 1 = liquid feces and 5 = extremely hard feces (Ireland-Perry and Stallings 1993).

d Fecal starch was determined using the K-TSTA kit (Megazyme, Lansing, MI, USA; method 996.11; AOAC 1986), while fecal N was determined according to the Kjeldahl method (method 988.05; AOAC 1995).

### Health observations

Table 6 reports the health observations of the steers during the experimental period. No differences were reported (*P* ≥ 0.12) on BRD occurrence, BRD without fever, other adverse health events, and number of BRD treatments to regain health. On the other hand, overall morbidity tended (*P* = 0.06) to be 25.7% lower, while occurrence of BRD + fever was reduced (*P* = 0.04) by 21.9%, in BAC-fed steers vs. CON (Table 6).

**Table 6:** Health observation results of crossbred beef steers receiving a high-moisture corn-based diet containing or not (*CON*; *n* = 14) a *bacillus*-based probiotic in the diet (*BAC*; *n* = 14)^a^^,^^b^.

Item	Treatments		SEM	*P-*value
	CON	BAC		
**Overall morbidity,^c^ %**	39.3	29.2	6.9	0.06
** Respiratory disease, %**	36.0	28.1	7.2	0.12
** With fever,^d^ %**	28.4	20.1	6.6	0.04
** Without fever, %**	7.6	8.0	1.8	0.68
** Others**	3.3	1.1	0.8	0.15
**Bovine respiratory disease**				
** One treatment to regain health, %**	24.8	21.2	5.9	0.40
** Two or more treatments to regain health, %**	10.5	6.9	2.1	0.23

a Treatments were offered daily from days 0 to 118. The BAC contained 2 g of *Bacillus licheniformis* 809 and *B. subtilis* 810 (6.4 × 10^9^ CFU/head per day; Bovacillus, Novonesis, Buenos Aires, Argentina) and was added to the mineral-vitamin premix.

b Steers were observed daily for signs of bovine respiratory disease (*BRD*).

c Calculated as number of animals showing an adverse health event divided by the number of animals enrolled into the study. In the table, overall morbidity = respiratory disease (%) + others.

d Steers classified as febrile when rectal temperature was > 39.5°C.

**Figure 1:**
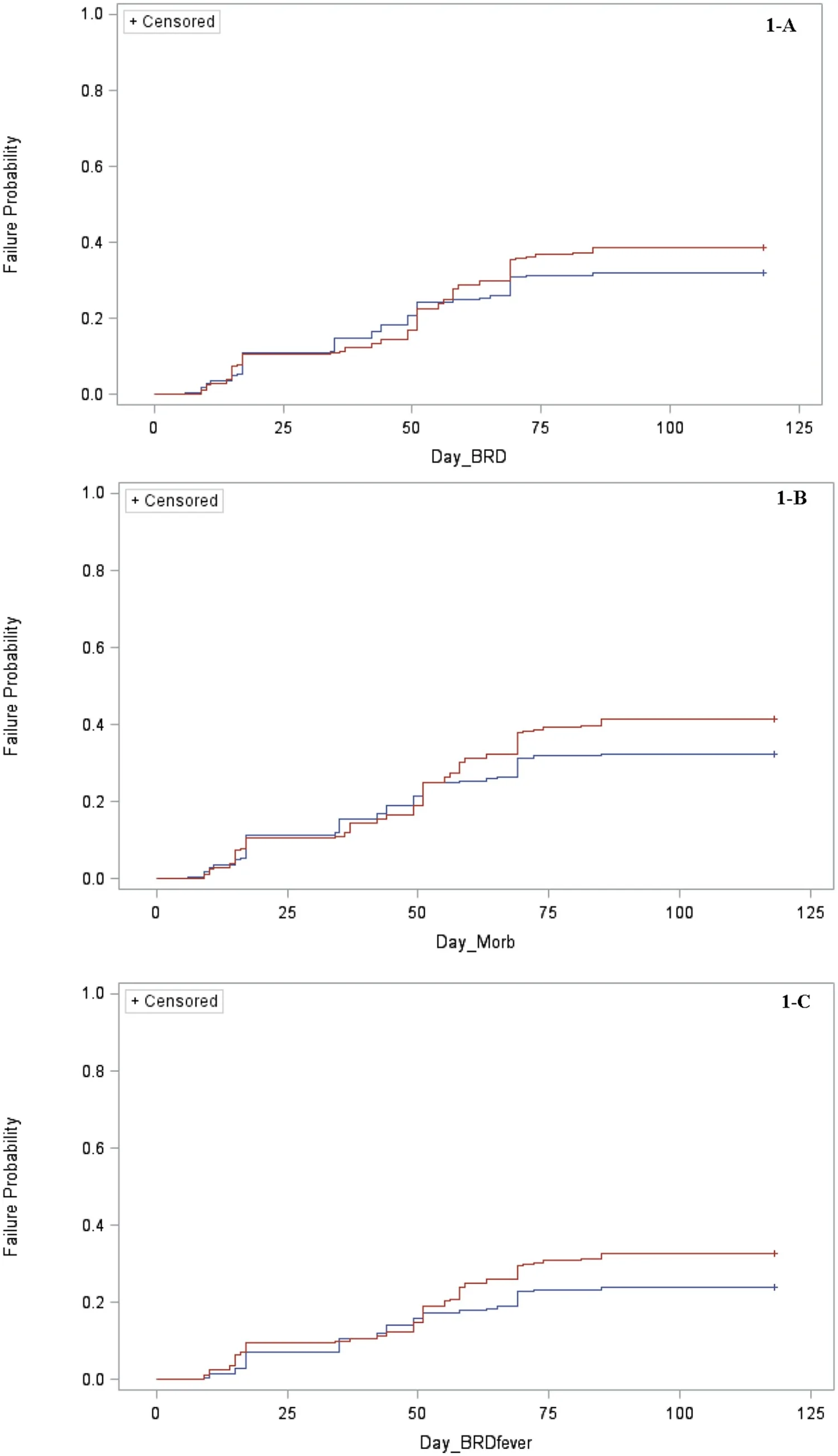
Temporal dynamics for overall bovine respiratory disease (*BRD*; A), overall morbidity (B), and BRD with fever occurrence (C) of crossbred beef steers receiving a high-moisture corn-based diet containing or not (*CON*; *n* = 14; red line) a *Bacillus*-based probiotic in the diet (*BAC*; *n* = 14; blue line).

Supplementing BAC did not alter the time-to-first event for overall BRD (log-rank; *P* = 0.22; 25th percentile at day 58 and 63 for CON and BAC, respectively; Fig. 1A) or general morbidity (log-rank; *P* = 0.12; 25th percentile at day 58 and 63 for CON and BAC, respectively; Fig. 1B). However, non-parametric survival analysis revealed a statistical trend (log-rank; *P* = 0.08) toward altered disease dynamics for steers showing BRD with fever, where BAC feeding delayed reaching 25% cumulative fever incidence by 26 d vs. CON (d 85 vs. d 59; Fig. 1C).

### Carcass traits

Table 7 reports the carcass traits of the steers fed either CON or BAC during the feedlot period. At slaughter, steers fed BAC had heavier (*P* = 0.05) carcasses than (3.5 kg or 1.2%) CON counterparts, but no differences were observed on dressing percent (*P* = 0.93; Table 7). The carcass-adjusted final BW, ADG, and GF were greater (*P* ≤ 0.05) for BAC-fed steers. No treatment effects were observed for ribeye area or backfat thickness (*P* ≥ 0.32), while *B. femoris* fat thickness tended to be 25.6% greater (*P* = 0.07) for BAC (Table 7).

**Table 7:** Carcass traits of crossbred beef steers receiving a high-moisture corn-based diet containing or not (*CON*; *n* = 14) a *bacillus*-based probiotic in the diet (*BAC*; *n* = 14)^a^^,^^b^.

Item	Treatments		SEM	*P-*value
	CON	BAC		
**Hot carcass weight, kg**	286.7	290.2	3.26	0.05
**Dressing percent**	59.5	59.6	0.26	0.93
**Carcass-adjusted final BW,^c^ kg**	494	500	5.6	0.05
**Carcass-adjusted ADG, kg**	1.40	1.45	0.05	0.05
**Carcass-adjusted gain to feed, kg/kg**	0.147	0.152	0.002	0.04
***Longissimus dorsi* area, cm** ^2^	77.1	74.8	1.92	0.35
**Backfat thickness, mm**	7.91	8.39	0.33	0.32
***Biceps femoris* thickness, mm**	8.60	10.8	0.80	0.07

a Treatments were offered daily from days 0 to 118. The BAC contained 2 g of *Bacillus licheniformis* 809 and *B. subtilis* 810 (6.4 × 10^9^ CFU/head per day; Bovacillus, Novonesis, Buenos Aires, Argentina) and was added to the mineral-vitamin premix.

b Sodium monensin was added to the mineral-vitamin premix of both treatments throughout the experimental period to ensure a dosage of 36 mg/kg.

c Carcass adjusted to 58% dressing percent, following industry guidelines in Argentina.

## Discussion

This study was designed to evaluate the effects of feeding a *Bacillus*-based probiotic containing *Bacillus licheniformis* 809 and *B. subtilis* 810 on feed intake, growth rates, adverse health event, and carcass traits of crossbred beef cattle consuming a high-moisture corn-based finishing diet (48% high-moisture corn; DM basis). It is noteworthy mentioning that the diets used herein also contained sodium monensin, an ionophore that has been extensively added to feedlot diets in North and South America (Vasconcelos and Galyean 2007; Samuelson et al. 2016; Monsalve and Millen 2025). Moreover, given the extensive literature on *B. licheniformis* 809 and *B. subtilis* 810 reporting the stability, modes of action, and performance of beef and dairy cattle (Cappellozza et al. 2025), no other study evaluated the effects of combining BAC, high-moisture corn, and sodium monensin on performance, health status, and carcass traits of feedlot cattle during finishing. In dairy cattle, apparent total-tract DM, organic matter, CP, NDF, and ether extract digestibility increased when *B. licheniformis* 809 and *B. subtilis* 810 were added to a TMR containing 2.3% high-moisture corn, but not when 11.4% high-moisture corn was added (Juckem et al. 2025), indicating a potential effect of dietary inclusion of high-moisture corn in the efficacy of a probiotic containing *B. licheniformis* 809 and *B. subtilis* 810. Hence, the effects of BAC in feedlot diets containing a greater proportion of high-moisture corn should also be evaluated, as high-moisture corn is a common grain processing method reported in North American surveys, while sodium monensin is the most used feed additive in feedlot settings (Vasconcelos and Galyean 2007; Samuelson et al. 2016).

The effects of *Bacillus*-based probiotics on performance of feedlot cattle have been variable over the available literature. In feedlot receiving cattle (45 days), supplementation of a *Bacillus*-based probiotic with yeast and monensin tended to increase BW gain when compared with the combination of tylosin and monensin, but did not differ from the *Bacillus subtilis* and yeast alone (Colombo et al. 2021). These authors also reported that feeding *B. subtilis* and yeast reduced the number of steers requiring a second intervention for BRD, mortality rates, and the number of animals removed from the study (Colombo et al. 2021), highlighting the health-supportive effects of *Bacillus*-based probiotics in feedlot cattle. Accordingly, *B. subtilis* PB6 supplementation reduced the first and second treatments for BRD, the cost of antimicrobial treatments, and increased live performance (final BW, ADG, and DMI) during the 56-day feedlot receiving period, while in the finishing period these effects were no longer significant and no further effects were observed on carcass traits (Smock et al. 2020). In another study, *B. subtilis* PB6 feeding for 140 days tended to decrease ADG, final BW, and % of carcasses classified as Low Choice (Smith et al. 2021). Other studies demonstrated variable results on health and performance when *B. subtilis* PB6 was offered to feedlot cattle (Word et al. 2022; Ryan et al. 2023). Previous authors suggested that the benefits of *Bacillus*-based probiotics on performance and health of feedlot cattle might be linked to the stress response modulation of the herd (DeHaan et al. 2024), which might explain the variation in the results. Moreover, the dose, species, strain, feeding management, and breed of animals (Krehbiel et al. 2003; McAllister et al. 2011; Santano et al. 2020) might also pose a challenge when evaluating the broader available literature on *Bacillus*-based probiotics for feedlot cattle. Hence, for a better understanding of the effects of a specific or a combination of microorganisms, the discussion herein will focus on the published results of a probiotic mixture containing *B. licheniformis* 809 and *B. subtilis* 810.

The improvements in overall ADG (+ 50 g) and final BW (+ 6.0 kg) are novel, as previous studies with BAC have not reported significant effects on both of these traits (Dias et al. 2022; Cordeiro et al. 2024). The dietary starch level in the previous studies ranged from 33% to 50%, being within the level reported herein; however, grain processing (ie high-moisture or steam-flaking) techniques were not used in the studies from Dias et al. (2022) or Cordeiro et al. (2024), as both fed only dry ground corn. In agreement with the previous studies, FE was positively impacted by BAC during the first 35 days of the study (+ 8.4%), but also during the entire experimental period (118 days; + 2.9%). Among the modes of action potentially leading to these results are the health-supportive effects (Mackey et al. 2024, 2025; Cappellozza et al. 2025), as well as enzymatic production (cellulases, xylanases, lipases, proteases, and amylases) and activity by *Bacillus* spp. (Schallmey et al. 2004; Elshaghabee et al. 2017; Luise et al. 2022) and, more specifically, the combination of *B. licheniformis* 809 and *B. subtilis* 810. Dry matter, NDF, and starch in vitro degradation have increased when BAC was added to single forage-based substrates, commercial dairy TMRs, and cereal grains (Pan et al. 2022; Cappellozza et al. 2023). In beef cow receiving a 9% CP mixed-grass hay and 0.5 kg dried distillers’ grains with solubles, BAC supplementation increased apparent total-tract DM and CP digestibility, while also trending to increase NDF and ADF digestibility (Limede et al. 2025). Lag time has been reported to be shorter when BAC was fed in comparison to a control or diets supplemented with ionophores, including sodium monensin (Da Silva et al. 2026; Limede et al. 2026), likely suggesting that digestion of the nutrients start earlier when BAC is added in the diet. Beef heifers fed a low-quality bermudagrass (*Cynodon dactylon*) hay with 1.0 kg concentrate containing BAC had greater in situ hay DM and NDF disappearance rate and effective degradability, while also having greater total-tract DM, CP, and NDF digestibility (Souza et al. 2026). Interestingly enough, subtle changes in the rumen microbiota profile were reported by Limede et al. (2025) and Souza et al. (2026), suggesting that the direct enzymatic effect of BAC seems to be playing a major role in these nutrient digestion results. Lastly, Cordeiro et al. (2024) observed lower fecal starch, likely caused by the reduced DMI, in BAC-fed bullocks receiving a feedlot diet containing 50% starch. Altogether, BAC is effective in the rumen environment by promoting nutrient degradation (Gresse et al. 2025), which in turn leads to a better overall nutrient utilization, growth, and efficiency in feedlot cattle (Vander Pol et al. 2008; Owens et al. 2016).

In the current study, no treatment effects were observed in DMI, an opposite result from Cordeiro et al. (2024). These latter authors reported a 3.1% lower DMI in crossbred feedlot cattle consuming a 50% starch finishing diet containing BAC. However, Cordeiro et al. (2024) fed finely ground corn (mean particle size = 1.0 mm), which likely was better utilized in the rumen of bullocks fed BAC, yielding greater propionate proportions, resulting in a trend of lower fecal starch, but also reducing the DMI likely caused by the hepatic oxidation of propionate (Allen et al. 2009). Supporting our results, Dias et al. (2022) did not report changes in DMI of beef steers fed a 33% starch diet, while rumen propionate molar proportion increased when BAC was fed. In the current study, no treatment effects were observed on either fecal starch or N levels at two different timepoints. However, the lack of effects on DMI and fecal nutrient levels might be related to the greater availability, digestion, and utilization of the high-moisture corn in the rumen, potentially limiting the effects of BAC. In a dairy cow study, BAC did not impact starch digestibility when high-moisture corn was added to the diet (Juckem et al. 2025), while total-tract NDF digestibility was greater in transition cows fed BAC (Nicola et al. 2026). Nonetheless, another important mode of action of BAC might be related to the host-related health-supportive actions (Cappellozza et al. 2025). Hence, it can be speculated that the offering of more ruminal fermentable substrates might have also altered the rumen and lower gut microbiota profile, as described by Pickett et al. (2022). This change could have challenged the lower gut and the different gut-related health effects of BAC might have played a role in maintaining an adequate microbial balance, oxidative stress status, and a proper integrity of the intestinal epithelial cells (Boll et al. 2024; Cordeiro et al. 2024; Capern et al. 2025, 2026; Cappellozza et al. 2025) that ultimately positively impacted the performance of the animals.

The relatively low observed energy values herein may be partially explained by the starch fecal levels, cattle biotype, and environmental conditions. In the tropics, corn hybrids used are generally of flint-type endosperm, which are more resistant to enzymatic attack when compared with dent-type hybrids (Correa et al. 2002; Kaczmarek et al. 2014). Flint-type endosperm is positively associated with grain hardness, density, and percentage of zein proteins involving starch granules, which can impair starch degradation (Giuberti et al. 2013). Recently, González et al. (2026) reported lower gas production (mL/g of DM) and fractional rate of gas production (per h), while lag time was higher in corn hybrids from Argentina vs. corn from the U.S. (soft kernel). In a feedlot study feeding roughly 79% of steam-flaked flint corn to finishing cattle, fecal starch level was 7.8% (Marques et al. 2016), which is comparable to our results. In the same study, when whole-flint corn was offered at different levels with sugarcane bagasse, fecal starch levels ranged from 26.7% to 32.3% (Marques et al. 2016). Regarding the environmental conditions and cattle biotype, the current study was conducted during the spring and summer in the Southern hemisphere with *Bos taurus*-influenced cattle, while THI was greater than 72 in approximately 45% of the experimental days (data not shown). Heat stress impacts the energy requirements of the animals, and even more in *Bos taurus* breeds, thereby decreasing the net energy partitioned for growth (NASEM et al. 2016). Furthermore, these animals did not receive anabolic implants or beta-agonists, and were castrated males, which inherently reduces energy utilization efficiency compared to the standard performance baselines often assumed in tabular NASEM et al. (2016) equations. Contrary to Dias et al. (2022), BAC feeding increased the observed to expected net energy ratios (for maintenance and gain) from days 0 to 35 and 0 to 118, likely a result of the greater live feedlot performance; yet both underperformed (ratio < 1), also indicating a lower than expected feedlot performance. In crossbred *Bos taurus* steers fed a corn silage-based diet for 84 days, Podversich et al. (2025) did not observe differences in energy efficiency between a mixture of essential oils and BAC and the same mixture with the addition of monensin. However, it is noteworthy mentioning that a treatment containing BAC alone was not evaluated by Podversich et al. (2025). It can be speculated that the differences between diets herein and the previous studies (Dias et al. 2022; Podversich et al. 2025) might partially explain the different results in efficiency of energy utilization.

The current lack of treatment effects on BRD incidence agrees with previous authors evaluating the supplementation of a probiotic containing *B. licheniformis* 809 and *B. subtilis* 810 to high-risk grazing cattle (Mackey et al. 2024). Nonetheless, overall removals and mortality + removals were reduced when the same *Bacillus*-based probiotic was fed (Mackey et al. 2024). In the current experiment, the occurrence of BRD and fever cases was lower, while overall morbidity tended to be lower in steers fed BAC vs. CON, suggesting a positive effect of the current probiotic mixture in the modulation of the immune response. The increase in rectal temperature is a defensive mechanism of the body when facing pathogenic and non-pathogenic challenges (Carroll and Forsberg 2007; Cooke 2017), while the increase in body temperature also increases the metabolism and nutrient requirements of the herd (Kluger and Rothenburg 1979; NASEM 2016). Recently, Mackey et al. (2025) reported lower rectal temperature in beef heifers offered BAC when mixed in a supplement and following the administration of a vaccine or lipopolysaccharide (*LPS*) challenge. Moreover, heifers fed BAC also had an alleviated innate immunity response, characterized by reduced plasma concentrations of tumor necrosis factor-alpha (*TNF-a*), faster resumption of feed intake, and reduced plasma concentrations of pro-inflammatory markers that were triggered by the LPS administration, specifically (Mackey et al. 2025). In cattle, the initial steps of an inflammatory response include a febrile response and synthesis of proinflammatory cytokines, such as TNF-a, which then stimulate hepatic synthesis of acute-phase proteins (Carroll and Forsberg 2007). These reactions will, in turn, impair cattle performance by increasing basal metabolism, stimulating tissue catabolism, depressing appetite, and altering overall behavior (Cooke 2017). Hence, the beneficial effects on the occurrence of fever + BRD cases suggest a health-supportive effect of BAC, likely positively impacting the metabolism, nutrient requirements, and performance results reported herein.

At slaughter, BAC feeding increased HCW by 3.5 kg, carcass-adjusted BW, ADG, and FCR, while also trending to increase *Biceps femoris* fat thickness. The increased carcass performance corroborated the live performance results of the animals, indicating that BAC feeding did not affect dressing percentage that could impact hot carcass weight. Previous studies in feedlot cattle reported greater feed efficiency following BAC feeding, but failed to report similar results in hot carcass weight or carcass traits (Dias et al. 2022; Cordeiro et al. 2024). The trend of BAC having greater *B. femoris* fat thickness vs. CON suggests a modulation of the rumen fermentation pathway that either increased the total volatile fatty acid (*VFA*) production in the rumen or a shift in the proportion of the individual VFA (Dias et al. 2022; Souza et al. 2026), as VFAs play a key role in fat synthesis among the different adipocyte depots in ruminants (Nayananjalie et al. 2015). Nonetheless, additional studies are warranted to understand the underlying effects and mechanisms of BAC on specific carcass traits, if any.

It is worth mentioning that the live and carcass trait results were observed in high-starch diets containing sodium monensin. Sodium monensin has been cited as the most used feed additive in North and South American feedlot diets (Vasconcelos and Galyean 2007; Samuelson et al. 2016; Monsalve and Millen 2025); hence, it makes sense to evaluate other feed additives in the presence of this ionophore in the diet. Monensin acts in the rumen by selectively inhibiting gram-positive bacteria, favoring the growth and activity of gram-negative bacteria which, in turn, alters the products of ruminal fermentation and rumen energetic efficiency (Russell and Strobel 1989; Marques and Cooke 2021). In beef cattle, Duffield et al. (2012) reported that monensin improves feed efficiency by 6.4% when compared with animals not receiving this ionophore, primarily due to a reduction in DMI (3%) and slightly improvement on ADG (2.5%) of the herd. To our knowledge, this is the first study evaluating the effects of BAC alone in feedlot diets containing sodium monensin and the positive results on live performance and carcass-adjusted traits also demonstrate the efficacy of BAC under this conventional dietary feedlot setting. In fact, the different, and perhaps complimentary, modes of action in the rumen and lower gut might explain the current results. However, additional studies are warranted to understand the potential synergistic effects of BAC and sodium monensin on rumen function, nutrient degradation, health, and fermentation patterns of beef animals consuming high-concentrate, high-starch finishing diets.

## Conclusion

In summary, feeding a probiotic mixture containing *Bacillus licheniformis* 809 and *Bacillus subtilis* 810 to feedlot cattle consuming a high-starch diet containing sodium monensin did not impact feed intake, but improved overall average daily gain (+ 50 g), feed efficiency (+ 50 g gain/kg of feed consumed), final BW (+ 6 kg), efficiency of energy utilization, and carcass weight (+ 3.5 kg) when compared with cohorts that were not offered the *Bacillus*-based probiotic. Feeding the *Bacillus*-based probiotic did not alter the total BRD prevalence; however, it decreased the occurrence of respiratory disease accompanied by fever and tended to decrease overall morbidity cases. Nonetheless, additional studies are further warranted to understand the metabolic effects of *B. licheniformis* 809 and *B. subtilis* 810 feeding to heat-stressed cattle, as well as the rumen and metabolic effects of combining sodium monensin and the *Bacillus*-based probiotic tested herein.

## List of abbreviation

ADF: Acid detergent fiber
ADG: Average daily gain
BAC: Treatment group fed the *Bacillus* spp.
BW: Body weight
CON: Treatment group fed the control diet
CP: Crude protein
DFM: Direct-fed microbial
DM: Dry matter
DMI: Dry matter intake
DP: Dressing percent
EG: Energy gain
FE: Feed efficiency
GF: Gain to feed
HCW: Hot carcass weight
N: Nitrogen
NDF: Neutral detergent fiber
NE: Net energy
NE_g_: Net energy for gain
NE_m_: Net energy for maintenance
TMR: Total mixed ration
TDN: Total digestible nutrients
